# Air Ambulance Contracting and Reimbursement and the No Surprises Act

**DOI:** 10.1001/jamanetworkopen.2026.6183

**Published:** 2026-04-10

**Authors:** Erin Duffy, Bich Ly, Erin Trish

**Affiliations:** 1 Leonard D. Schaeffer Center for Health Policy & Economics, University of Southern California, Los Angeles; 2 Sol Price School of Public Policy, University of Southern California, Los Angeles; 3 Alfred E. Mann School of Pharmacy and Pharmaceutical Sciences, University of Southern California, Los Angeles

## Abstract

This cross-sectional study assesses trends in air ambulance service network status composition, in-network negotiated prices, and out-of-network allowed amounts prior to and in the first year of implementation of the federal No Surprises Act.

## Introduction

Air ambulances are used by approximately 1 in 4000 commercially insured patients annually when distances, medical urgency, and other conditions prohibit ground travel.^1^ Historically, out-of-network air ambulance organizations could bill commercially insured patients for the balance between their charges and the insurer’s allowed amount, yielding surprise bills averaging approximately $20 000.^2,3,4^ Surprise billing for air ambulance services was banned by the federal No Surprises Act (NSA), passed by Congress in December 2020 and implemented in January 2022. The NSA limits cost sharing for out-of-network services to the amount that would be owed for an in-network service based on median contracted rates in 2019, adjusted for inflation, measures known as qualifying payment amounts. The NSA’s independent dispute resolution process also includes qualifying payment amounts as benchmarks, among other factors, when arbitrating insurer–health care professional payment disagreements. This study describes air ambulance service network status composition, in-network negotiated prices, and out-of-network allowed amounts during the years leading up to the passage of the NSA and the first year of its implementation.

## Methods

This cross-sectional study analyzed Health Care Cost Institute’s commercial insurance claims for rotary activation services from 2012 through 2022 for patients younger than 65 years (see the eMethods in Supplement 1 for sample attrition details). Annual proportions of in-network and out-of-network services were calculated. Annual mean (SD) allowed amounts were calculated separately for in-network and out-of-network services. Allowed amounts were computed for rural pickups, urban pickups, and all pickups combined to account for potential price differences by location type. As sensitivity analyses, mean (SD) allowed amounts were computed for the total transport episode: activation plus mileage. Additionally, ratios of commercial prices to Medicare allowable amounts were computed to account for potential geographic composition changes. The institutional review board of the University of Southern California approved the study and determined that it was not human participant research, and therefore informed consent was not required. Statistical analyses were performed from August 25, 2025, to November 24, 2025 using SAS, version 9.4, using nominal dollars. This report followed the STROBE reporting guideline.

## Results

**Figure 1.**
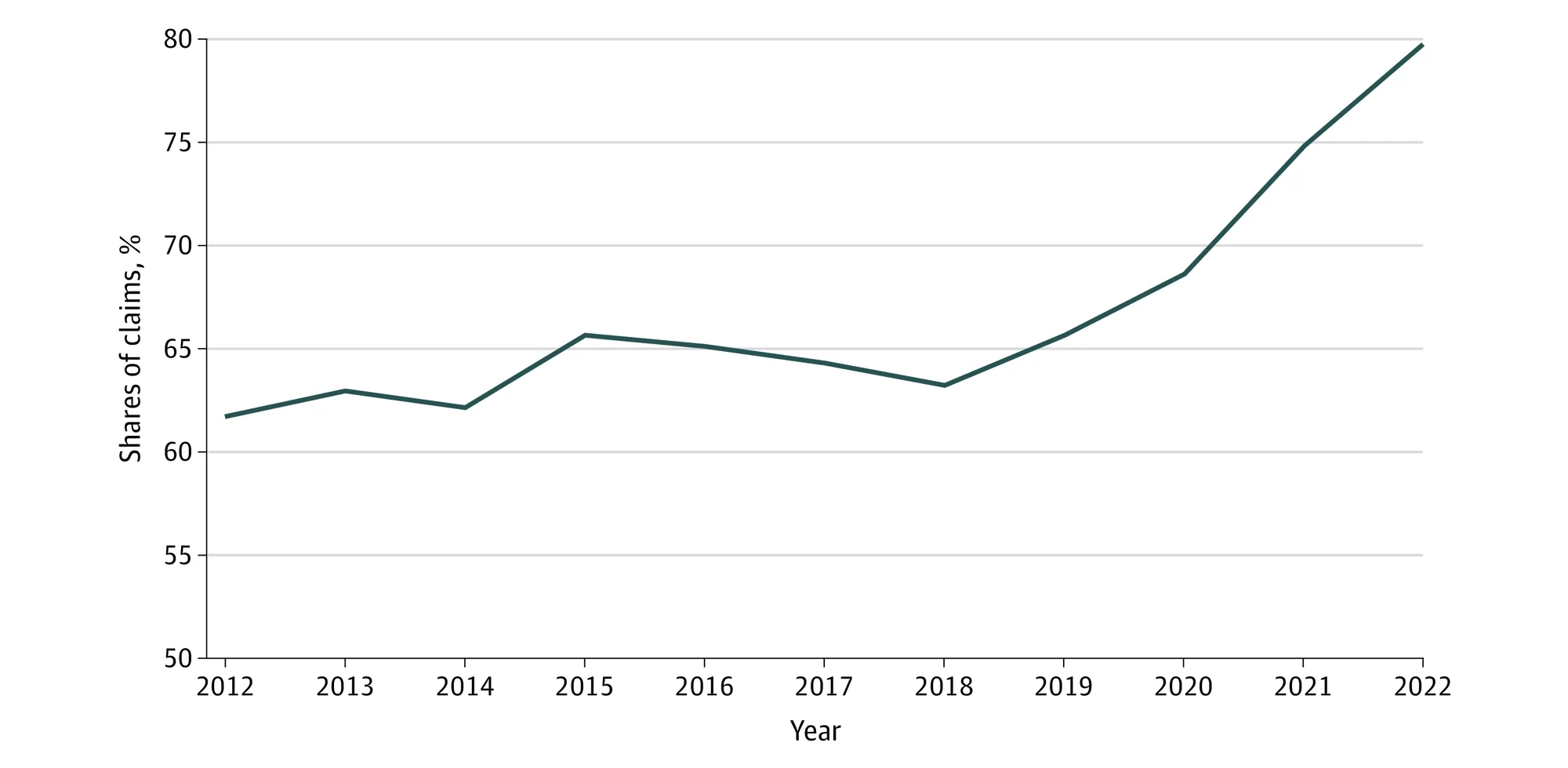
Line Graph of Trends in the Prevalence of In-Network Rotary-Wing Air Ambulance Services The 2012 to 2022 Health Care Cost Institute commercial claims data were analyzed.

The study sample included 122 545 rotary activation claims for 81 333 in-network and 41 212 out-of-network claims from 117 988 patients (63% men, 52.3% younger than 45 years, and 47.7% aged 45-64 years). The share of claims provided in network remained relatively stable from 2012 through 2019, fluctuated between 62% and 66%, and then increased in 2020 (69%), 2021 (75%), and 2022 (80%) (Figure 1).

**Figure 2.**
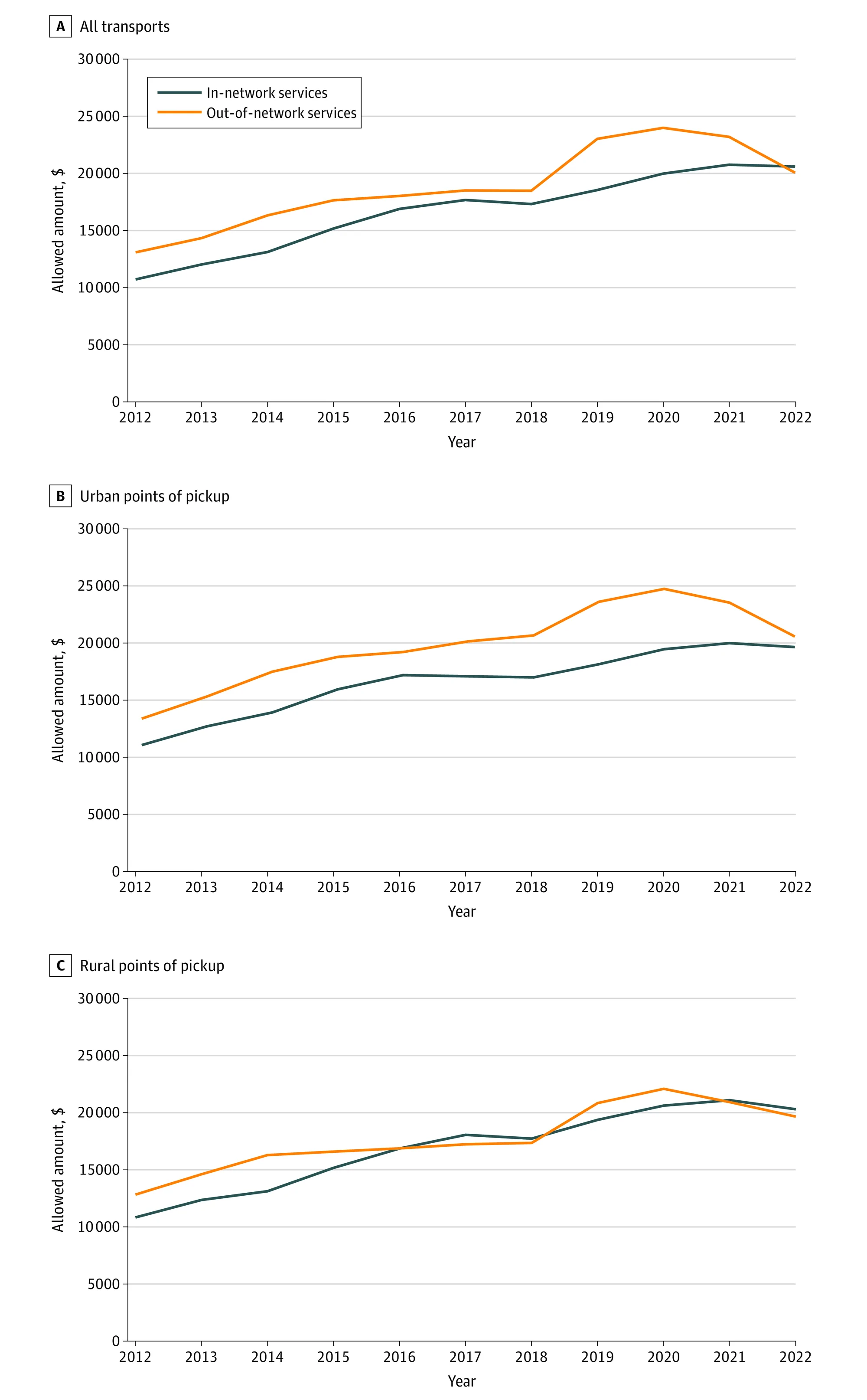
Line Graphs of Trends in Mean Base Allowed Amounts for In-Network and Out-of-Network Rotary-Wing Air Ambulance Services The 2012 to 2022 Health Care Cost Institute commercial claims data were analyzed.

Mean (SD) in-network allowed amounts increased from $10 722 ($5113) in 2012 to a peak of $20 754 ($10 449) in 2021, decreasing to $20 593 ($10 081) in 2022 (Figure 2A). Out-of-network allowed amounts increased from $13 091 ($5248) in 2012 to a peak of $23 978 ($14 398) in 2020 and then decreased in 2021 ($23 188 [$15 778]) and 2022 ($20 056 [$9750]). Similar patterns are observed within urban (Figure 2B) and rural (Figure 2C) pickups and in sensitivity analyses using allowed amount ratios to Medicare and incorporating mileage. Notably, across these sensitivity analyses, there is a more muted difference between in-network and out-of-network trendlines among rural claims.

## Discussion

This cross-sectional study of commercial insurance claims found that a decrease in allowed amounts coincided with the passage and implementation of the NSA, as well as a shift toward in-network services. These findings are consistent with insights from a 2024 study^5^ in which interviews with air ambulance organizations and insurers indicated that the NSA may have motivated new network contracts and put downward pressure on reimbursements. The market may evolve further as litigation about the implementation of the NSA continues.^6^ Limitations include potential limited generalizability to insurers outside the sample and a lack of causal evaluation.

## Notes

Supplement 1. **eMethods.** Analytical Sample Construction

Supplement 2.
Data Sharing Statement

## References

[1] Turrini G, Ruhter J, Chappel AR, De Lew N. Air ambulance use and surprise billing. Department of Health and Human Services. Assistant Secretary of Planning and Evaluation. Office of Health Policy. Issue Brief. September 10, 2021. HP-2021-HP-20. Accessed September 10, 2024 https://aspe.hhs.gov/sites/default/files/2021-09/aspe-air-ambulance-ib-09-10-2021.pdf

[2] Fuse Brown EC, Trish E, Ly B, Hall M, Adler L. Out-of-network air ambulance bills: prevalence, magnitude, and policy solutions. Milbank Q. 2020;98(3):747-774. doi:10.1111/1468-0009.12464 32525223 PMC7482379

[3] Chhabra KR, McGuire K, Sheetz KH, Scott JW, Nuliyalu U, Ryan AM. Most patients undergoing ground and air ambulance transportation receive sizable out-of-network bills. Health Aff (Millwood). 2020;39(5):777-782. doi:10.1377/hlthaff.2019.01484 32293925

[4] Garmon C, Chartock B. One in five inpatient emergency department cases may lead to surprise bills. Health Aff (Millwood). 2017;36(1):177-181. doi:10.1377/hlthaff.2016.0970 27974361

[5] Rasmussen PW, Duffy EL, Yardi I, . The Implications of the No Surprises Act on Contract Dynamics, Negotiations, and Finances: Perspectives From Key Stakeholders. RAND Corporation; 2024.

[6] No Surprises Act. Health care litigation tracker. O’Neill Institute Accessed September 10, 2025. https://litigationtracker.law.georgetown.edu/issues/no-surprises-act/

