# Error in Figure Title

**DOI:** 10.1001/jamanetworkopen.2026.40433

**Published:** 2026-10-05

In the Research Letter titled “Air Ambulance Contracting and Reimbursement and the No Surprises Act”^1^ published on April 10, 2026, the title of Figure 1 indicated that the graph showed trends in the prevalence of out-of-network rotary-wing ambulance services. In fact, the graph shows in-network services. This article has been corrected.^1^
