# Supplementary material for: Air Ambulance Contracting and Reimbursement and the No Surprises Act
Source: JAMA Netw Open. 2026 Apr 10;9(4):e266183. doi: 10.1001/jamanetworkopen.2026.6183 (PMC13069454; doi:10.1001/jamanetworkopen.2026.6183)
Supplement: Supplement 1. — eMethods. Analytical Sample Construction [file jamanetwopen-e266183-s001.pdf]

Supplemental Online Content

Duffy E, Ly B, Trish E. Air ambulance contracting and reimbursement and the No Surprises Act. *JAMA Netw Open*. 2026;9(4):e266183.
doi:10.1001/jamanetworkopen.2026.6183

eMethods. Analytical Sample Construction

This supplemental material has been provided by the authors to give readers additional information about their work.

© 2026 Duffy E et al. *JAMA Network Open.*

1. **eMethods.** Analytical Sample Construction

We identified approximately 149,000 rotary-wing air ambulance claims from 2012 to 2022 for commercially insured patients under 65 years old. The claims are identified using HCPC codes A0431 (base amount) and A0436 (mileage). Secondary, non-primary payer claims are excluded from our sample. We also exclude claims with 1) total allowed amount of zero or less, 2) missing base or mileage amounts, 3) other data anomalies such as inconsistent values for network status. There are approximately 122,500 claims in our sample after these exclusions. The table below summarizes our data sample and exclusion criteria.

eTable 1. Attrition Table

	# of Claims	% of Claims
Rotary-wing air ambulance claims for commercially insured patients under 65 years old	149,032	
Exclude claims that have a total allowed amount of zero or less	18,900	13%
Exclude claims with other data anomalies	7,587	5%
Total Exclusions	26,487	18%
**Sample for Analysis**	**122,545**	**82%**

Note: Other data anomalies include claims with missing claim lines for either base or mileage amount or inconsistent values for network status across claim lines.

Since the HCCI data does not include a geographic indicator for where an air ambulance pick-up occurred, we restricted our samples for rural and urban stratified analyses to a subset of interfacility transfers where we can identify the locations of the facilities from which the patients were transferred from[1]. For these claims, we identified the corresponding facility claims from which the patients were transferred from and used the

[1] We identified these interfacility transfer claims using modifier = HH. These claims represent 42% of our sample.

© 2026 Duffy E et al. *JAMA Network Open.*

facilities' locations as the proxy for the pick-up locations to determine whether it is an urban or rural point-of-pickup. Thus, the sample for rural and urban claims is only for inter-facility transfers, a subset of the full sample: 16,470 in-network urban point-of-pickup, 15,789 in-network rural point-of-pickup, 9,871 out-of-network urban point-of-pickup, and 9,158 out-of-network rural point-of-pickup.

© 2026 Duffy E et al. *JAMA Network Open.*
