# Supplementary material for: Air Ambulance Contracting and Reimbursement and the No Surprises Act
Source: JAMA Netw Open. 2026 Apr 10;9(4):e266183. doi: 10.1001/jamanetworkopen.2026.6183 (PMC13069454; doi:10.1001/jamanetworkopen.2026.6183)
Supplement: Supplement 2. — Data Sharing Statement [file jamanetwopen-e266183-s002.pdf]

Data Sharing Statement

Duffy. Air Ambulance Contracting and Reimbursement and the No Surprises Act. *JAMA Netw Open*. Published April 10, 2026. doi:10.1001/jamanetworkopen.2026.6183

Data
Data available: No

Additional Information
Explanation for why data not available: Data are available through Health Care Cost Institute.
